# Digital Competencies for Nurses: Tools for Responding to Spiritual Care Needs

**DOI:** 10.3390/healthcare10101966

**Published:** 2022-10-08

**Authors:** Daniel Burgos, Aída López-Serrano, Stefania Palmisano, Fiona Timmins, Michael Connolly

**Affiliations:** 1 Research Institute for Innovation & Technology in Education (UNIR iTED), Universidad Internacional de La Rioja (UNIR), 26006 Logroño, Spain; 2 Faculty of Humanities, Universidad Internacional de La Rioja (UNIR), 26006 Logroño, Spain; 3 Department of Culture, Politics and Society, University of Turin, 10241 Torino, Italy; 4 School of Nursing, Midwifery and Health Systems, University College Dublin, D04 V1W8 Dublin, Ireland; 5 Education & Research Centre, Our Lady’s Hospice & Care Services, D6W RY72 Dublin, Ireland

**Keywords:** spirituality, digital competence, online learning, healthcare practitioners, health systems, nursing

## Abstract

Users show a growing interest in expanding the implementation of digital tools as a support of technical and management issues in healthcare. This medical care has focused on telemedicine but does not include the recognition of needs as an important part of patient-centred healthcare. Nurses interact with patients at critical times in their life journeys, including birth and death, which are historical events linked with religious beliefs. Furthermore, large migration flows have led to multicultural societies in which religion and spirituality are experienced in distinct ways by different people. Finally, most healthcare professionals lack the proper skills to handle the spiritual needs of their patients, especially for core and digital competences. This article shows the results of qualitative research applying as a research tool an open-ended questionnaire, which allows detecting the educational needs for nurses’ interventions aimed at providing spiritual support to their patients using digital tools. The results obtained reveal that nurses need education and training on fundamental spiritual concepts and digital competencies to meet the multiple demands of their patients’ spiritual needs. Finally, we present an open digital educational proposal for the development of competencies for nurses and other health professionals to provide spiritual care with the support of digital tools.

## 1. Introduction

Spirituality is a polysemic concept to which heterogeneous and sometimes elusive and ill-defined meanings are attributed. As a component of the human being seen as a totality, spirituality is innate. It refers to the depth of the human being and to his emotions. It can be linked to or distinguished from organized religion, and it implies dynamism, movement, development, and research [1].

Different types of spiritualities can be distinguished: God-oriented spiritualities, immanent world-oriented spiritualities that emphasize ecology, and humanistic spiritualities that value human potential and success in the world. All these different forms of spirituality urge a search for connection with something greater than the individual ego, namely the soul, others, nature, and a sense of mystery.

Spirituality is acknowledged as a critical aspect in the provision of holistic healthcare [2,3,4]. There is solid evidence that many patients and their relatives are spiritually in need during the provision of healthcare [5] and that actions taken in this regard are cost-effective [6,7]. The provision of patients’ spiritual and religious needs can be beneficial, helping in recovery or facilitating peaceful death [8]. To this extent, spirituality is a quest for compatibility and meaning, divine existence, and belonging [9].

The spiritual care offered by nurses aims to achieve greater effectiveness through the development of competencies in the various dimensions of spiritual care. Studies such as the one conducted in China in 2020 show how nurses develop different levels of competencies related to self-efficacy, training in their first degree, and experience in caring for terminally ill patients [10]. They conclude that there is a need to improve nurses’ competence to meet the spiritual care needs of patients, using appropriate learning methodologies.

The competences of attention to spirituality must focus on the characteristics of the individual person, who can be spiritual without being religious, but there exists an assumption that religious people are spiritual [11], as being religious is the outward practice of spirituality in organized religion. Spiritual care is interpreted as taking care of patients’ spiritual fears, struggles, and worries; supporting their spirituality and listening to their spiritual needs [12]. Devotion, religious coping, and spirituality are linked with a growing life satisfaction as well as quality of life [13] in connection with chronic illnesses [14,15]. Religious coping and spirituality are essential when someone gets bad news or when facing serious clinical diagnoses or tragic events [2]. Spirituality is also a means of offering hope to patients with chronic illnesses as an essential perspective for well-being [16]. At the end of life, the provision of spiritual support alleviates suffering and is greatly appreciated by most patients and families [17,18,19,20]. Spirituality is also a source of support when moral injury is committed [21]. These multiple facets of spirituality and the attention to it require a methodology that includes different forms and flexible approaches that are appropriate for the diverse patients’ needs as well as for nurses and other practitioners in healthcare that care for them. To this end, the digital environment offers tools for the development of appropriate competencies.

### 1.1. Background

The COVID-19 pandemic brings a cause–effect impact on the traditional healthcare context, forcing professionals to use—in a generalized manner and in a short space of time—remote intervention technologies that were previously used only occasionally in specific situations. This rapid adaptation has favoured new health technologies, which have become an essential element in the daily life of society. The pandemic has also led healthcare professionals to become aware of the critical role of spiritual support needs [22,23]. During the hardest times of the pandemic, there were creative efforts by healthcare chaplaincy services, among others, to help patients in distress and in isolation [22,24]. Restrictions imposed on hospitalized patients inflicted unprecedented stress that was further pronounced by isolation. Many patients sought support from spiritual and religious sources [25], and chaplains reported an increase in demand for their services [26]. The need for intensive and creative responses to meet these challenges was evident throughout the world [21]. Healthcare professionals made a significant effort to create a dignifying space where the cognitive and physical decline process would be convenient [26]. To this end, integrated and person-centred health services faced with the need to pay special attention to the needs and preferences of the individual have found it necessary to incorporate an innovative method to improve their ability to respond to the new challenges emerging in the field of emotional, social, and spiritual care in end-of-life processes. This method has taken advantage of the beneficial opportunities offered by technology.

The investigation of the role of nurses and the providing of spiritual care became widespread [27]. However, competent knowledge in providing spiritual care was often suboptimal given their poor education and training in this task.

In healthcare settings, the chaplain has traditionally been accepted as the person with the knowledge and experience to give spiritual care with pastoral and religious/faith-based services [28]. Indeed, healthcare chaplaincy support has demonstrated beneficial effects on patients, helping them navigate important life journeys and interpret their experiences. However, healthcare disciplines today are no longer monopolized by single practitioners, and spirituality support is increasingly becoming a multidisciplinary responsibility [29]. There is a need to coordinate and collaborate in all efforts to deliver pastoral needs [22,29]. New interactions between different actors of spiritual care support have raised several questions:

- Should spiritual care fall primarily (or exclusively) within the remit of traditional and formal practitioners, i.e., healthcare chaplains?
- Should healthcare professionals such as doctors and nurses be involved in providing spiritual support?
- Is spiritual care a private, individual matter that should remain untouchable and under the responsibility of the patient, family, or friends [30]?
- Are digital competencies a useful tool for providing care for patients’ spiritual needs?

These questions point to essential issues about the backbone that supports religious and spiritual activity and the role that professionals of healthcare may play [21,31]. There is demand for spiritual care services [4], but there is a lack of evidence for nurses’ spiritual care education and training [4,32,33]. There is also minimum guidance for assessing and providing spiritual care in practice. Moreover, nurses are sensitive in providing spiritual care, which increases patients’ well-being and satisfaction [34,35].

### 1.2. Nurses and the Provision of Spiritual Care

Nurses perform some spiritual care that is associated with longstanding international and national standards—for instance, the care reported by NANDA International (Nanda-I) [35] and the Royal College of Nursing [36]. Recently, there has been a development of specific competencies in the European context for midwives and nurses [4,32]. These competencies were designed under public funding provided by the Erasmus Plus Programme thanks to a project that built on innovation, compassionate care, and education to support the development of competences in spiritual care by midwives and nurses [32]. This helped provide clear guidance for nurses in supporting patients’ spirituality, mainly by increasing awareness of their spirituality. It also helped nurses to assess specific needs for spiritual care from patients and to address them properly [32,37].

According to the framework [32], nurses should identify spiritual needs and resources in order to plan, assess, and report interventions, outcomes, and processes in an effective way. Nurses should also be aware of their limitations and draw on expert resources when the need arises [38]. However, this does not imply that nurses should replace healthcare chaplains; rather, experts in healthcare, such as nurses, are more qualified to identify patients’ needs and to provide effective support. Further, they can re-route to other experts and services, such as chaplains, which could be beneficial as well.

In the past three decades, nurses have requested specific training on the topic (e.g., spiritual care) [4] though they undergo training even without standardized education. Pastrana et al. [39], along with other authors [40], claimed a need for immediate work with nurses since they reported a lack of specific competencies in spiritual care. Several other authors have noted the need for spiritual care education for nurses. Nurses believe that patients have spiritual needs, and these are being supported even without formal training [36], including through their own resources, often with digital tools through which they find guidance and information for their spiritual care interventions.

### 1.3. Digital Competencies for Nurses

The second phase of the project defines the digital competencies necessary for the continuous training of professionals providing spiritual care. For this purpose, the following learning areas were identified: (1) spiritual sense, (2) clinical environment, and (3) digital competences.

The assessment of whether nurses can be digitally competent in the care they provide in the social and healthcare field has been the subject of some studies, such as the one carried out by Konttila et al. [41]. The objective of this research was to systematically review studies focused on healthcare professionals’ competence levels in digitalisation. This systematic review determined the nurses’ level for each of the digital competencies and identified areas for improvement. Furthermore, there are some gaps in nurses’ digital competencies, according to Terry et al. [42] and Fernández-Llamazares et al. [43]. This last study showed that 72.4% of nurses access the Internet daily, but only 22.2% use it to search for information about their work every day. In terms of skills, most of the nurses considered it important to use secure channels to store and share information in digital environments, and the item with the lowest score was knowledge of creating and developing applications to generate content.

To address these needs for nurses to acquire digital competencies in the field of health, the strategic objectives in the Global Digital Health Strategy 2020–2024 [44] include advocating for people-centred health systems that are trained for digital health, and the proposed actions for health policy include developing training for professionals in digital health to improve literacy in eHealth, a term that defines the set of information and communication technologies (ICTs). These are tools used in the healthcare environment for prevention, diagnosis, treatment, and follow-up as well as in health management to save costs to the healthcare system and improve its efficiency. For this, it is necessary to carry out training programs based on the competencies that professionals need to acquire.

However, as these digital competencies for health professionals have only recently been defined, the specific competencies for spiritual care that nurses have to offer patients have not been specified. Research in this area is scarce, and the extent to which they have acquired competencies is not known. As such, the objective of this study is to determine nurses’ level for each of the digital competencies and to identify areas for improvement as well as examine the professional profile associated with the level of competence.

Professionals are acquiring an increasing number of competencies since, in the healthcare field, we can say that there is no service or aspect that remains outside their influence, for example, electronic medical records, telecare, appointments, service planning, patient follow-up, care, and healthcare management with technological solutions that help improve efficiency, safety, and patient experience.

ICTs are tools that reinforce and improve the healthcare model, and nurses in particular have begun to make intensive use of them. The main digital competencies that are considered useful and beneficial for nursing professionals are those related to:

- Searching for and sharing information with other professionals in the sector through different social networks;
- Sharing information with patients through social networks and content portals created ad hoc by health services;
- Creating their “own digital brand”, gaining followers, and interacting, which helps create a “digital reputation” that improves and increases professional opportunities and relationships with the sector;
- Creating their own content related to topics of their specialty, which are usually published through personal blogs or the blogs of health services—not only creating content that is shared for free but managing and growing the visibility of that blog, possibly working intuitively using the blog’s SEO, and disseminating content through digital channels;
- Adapting to and accepting new technological solutions implemented in hospitals that favour and improve both the health and quality of life of patients as well as aspects related to health management and the costs involved; planning tasks, resources, logistics, and personnel; researching and providing information for citizens in general and patients and families in particular; conducting prevention and awareness campaigns; conducting data measurement and analysis; ensuring privacy of patient data; and tending to and working with an increasingly empowered patient or “expert patient” who is informed and relates more and more through the digital environment.

ICTs in healthcare are more than present; they certainly pose a challenge, which is characterized by:

- Being able to “navigate” the amount of information that is generated and select valuable content;
- Teaching patients so that they also know how to select information;
- Attending to the sometimes-unnecessary demands and queries of patients, which are made in a context of immediacy;
- Managing important changes taking place in healthcare organizations with the implementation of management solutions, which in many cases leads to a change in work processes;
- Maintaining the privacy and security of patient data;
- Managing the compatibility of technology with face-to-face patient care.

### 1.4. Education for Spiritual Care in the Hospital Setting

To this extent, the Erasmus Plus Project “From Cure to Care: Digital Education and Spiritual Assistance in Healthcare” [45] is focused on the competence achievement on spiritual care by healthcare practitioners. In doing so, these professionals will be equipped to address the patients’ needs regarding existential crisis. The project promotes innovation, mostly in the undergraduate nursing curriculum, to provide this type of support through digital tools, with a focus on responding to the recent challenges of the COVID-19 pandemic.

**Figure 1:**
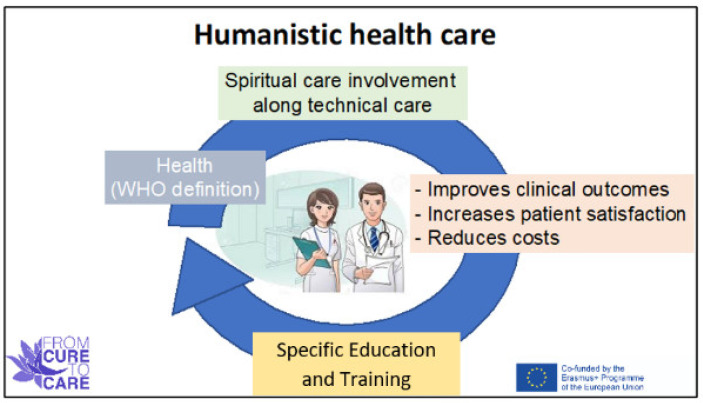
Achievement of humanistic healthcare by doctors and nurses.

The project “From Cure to Care” will develop an online learning program to support the achievement of two sets of competencies and keys for spiritual care. The program will help modify the nurses’ curriculum and keep up with their best practices [46]. Digital and religious/spiritual competencies, in a context of multiple cultures, are often absent from nursing curricula. These competencies will help nurses address spiritual and religious requests from patients. The implementation of this curriculum that includes digital competencies will be the primary contribution of the project in the field of digital education readiness in spiritual care. Various other experiences focused on online education and spiritual care and addressed to nurses and healthcare practitioners are encouraging [32,37]. We shall report phase 1 of “From Cure to Care” from the perspective of humanistic healthcare (Figure 1).

In this phase, it was required to produce state-of-the-art spiritual care facilities and practices to address gaps in adequate service provision and to prepare the educational program. It is crucial to establish the role that nurses play in the provision of spiritual care in order to better understand potential overlaps and different views amongst the countries of the project partners (i.e., Poland, Ireland, Italy, and Spain). This state-of-the-art also shows the way that healthcare practitioners work to support spiritual needs of patients, supported by digital tools. Finally, it will yield information to finalize the nursing curriculum towards the project’s goal.

## 2. Methodology

### 2.1. Research Objective

This research aims to identify the educational needs of healthcare professionals for the spiritual care of patients, which can be acquired through digital competencies. The research results will be applied to improve the nurses’ educational program for spiritual care thanks to an online learning course that will be implemented in the context of the aforementioned project.

### 2.2. Methods

The research method used was the case study, as it is the most appropriate in the process of inquiry, as well as the systematic analysis of the situation of professionals in the different countries of Europe. This method allows us to know in detail the circumstances, situations, and needs of the professionals who offer spiritual care in each of the five countries, offering a response per country.

The methodological approach used for the study was qualitative, focused on action research, which is understood as the study of spiritual care, to improve the quality of nurses’ learning and expand their professional competence.

The research paradigm is descriptive of the spiritual care situation in hospitals, according to their characteristics and resources. In addition, it is an interpretative paradigm based on the meanings of spiritual care action, which allows for understanding reality from the experiences and meanings of the nurses involved in this care. It also allows for knowing the intentions, beliefs, and motivations of the nurses.

### 2.3. Instruments Used for Data Collection

The research tool was a questionnaire with open-ended questions provided in a template (available under request to the corresponding author). The categories of analysis designed correspond to:

- Understanding the patients’ needs and considering the various cultures, beliefs, and religions;
- Providing spiritual care to all patients, including those of a minority faith;
- Assessing how to manage those needs;
- Using assessment tools to address spiritual well-being needs;
- Assessing and supporting support spiritual needs supported by digital tools.

Fieldwork experts developed a short form to gather this information from universities in the consortium, which produced data collection. The form is distributed in seven sections for a total of 33 questions. Three sections were designed out of a tool focused on healthcare resources and spiritual care and how institutions develop policies on the topic, considering context, facilities, and other inputs [47].

The other four sections were designed based on the outcomes of the EPICC project, which provided nurses with four core competences, namely identification of patients’ spiritual needs and resources; development of strategies for spiritual engagement; evaluate health outcomes; and documenting care statistics [4].

The researchers developed the collection form based on the competency framework produced by the EPICC project (2021) and other related field work [47]. Four meetings with international experts were required to produce the form. In particular, a competency framework formed part of the survey in this project. It provided a healthy guideline regarding spiritual care competencies where nurses serve as a skilful guide to care in healthcare settings.

### 2.4. Study Setting

This study provided a state-of-the-art of the resources and practices on spiritual care in four European countries (i.e., Italy, Poland, Ireland, and Spain, where two surveys were implemented). All this information remains under public domain and direct request to the authors [47].

### 2.5. Population and Sample

The study population corresponds to healthcare professionals, specifically nursing professionals, from four European countries: Italy, Ireland, Poland, and Spain. The sample selected were from four hospital centres in Italy, Ireland, Poland, and Spain, selected by each participating university partner of the project (From Cure to Care) to focus its analysis. The criteria for selecting the sample were that they should be nursing professionals in team management or managerial positions, and both men and women were considered to be necessary.

### 2.6. Data Processing

The information was collected in an Excel database common to the Erasmus Plus project “From Cure to Care”. For processing the information for each case, the information was collected and organized according to each of the categories of analysis. First, it was categorized according to each of the six sections of information. Second, a category was established for each of the items or questions asked. According to this categorization, valid information was selected for qualitative analysis according to the significance. For this purpose, the opinions and perceptions that occurred in the greatest number of cases were highlighted. Exceptional cases were also highlighted.

### 2.7. Operational Definitions

A skilful framework tool was objectively engaged in the collection of theoretical data [32,37], and key definitions and stipulations of spirituality were inherited from EPICC. In this context, the authors refer to spirituality as “the transformative aspect of human life that relates to the way persons (individual and society, community) experience, express and seek meaning, purpose and transcendence, and the way they connect to the moment, to self, to others, to nature, to the significant and/or the sacred”.

The European Association for Palliative Care (EAPC) adopted this definition. Moreover, the NHS Education Scotland integrated an adapted version. These adoptions worked to reflect well-being as well as illness [32]. The project stated a multidimensional approach of spirituality:

- There are empirically inclined challenges, such as responsibility and freedom, despair and hope, and others;
- There must be consideration of values and attitudes, such as culture and art, morals and ethics, and others;
- There are also religious doctrines, foundations, and guidelines, such as beliefs and practices, relationship with God, and others.

### 2.8. Data Analysis

The qualitative data analysis was systematic, following a sequence and order through the following phases: obtaining the information through interviews and capturing the information in order and coding the information by grouping the information obtained in four categories that concentrate the themes discovered: healthcare facility characteristics and spiritual care resources, intrapersonal spirituality, interpersonal spirituality, and spiritual care intervention and evaluation. These were assigned codes according to the units of meaning to the descriptive information compiled during an investigation: integration of the information relating the categories together with the theoretical foundations of the investigation.

## 3. Results

### 3.1. Healthcare Facility Characteristics and Spiritual Care Resources

**Figure 2:**
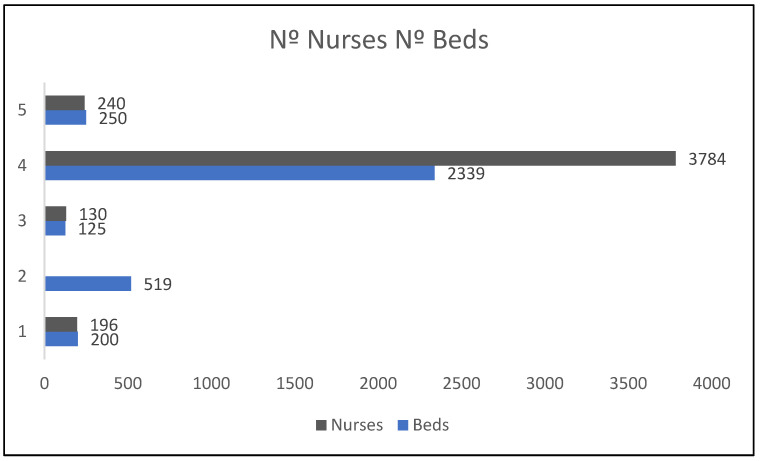
Comparison of number of nurses and number of beds.

Healthcare facilities correspond mainly to hospitals, with only one case being a hospice. In terms of size, they are generally between 196 and 2339 beds, distributed across rooms. Care is provided by 130 nurses for the smallest number of beds and 3784 nurses for the largest number of beds (Figure 2).

The main—and only—space for prayer in these hospitals is the Catholic chapels. However, the affiliation of the hospital centres to a religious community exists in only one case out of the five analysed.

All the religious services provided in the healthcare facilities analysed are daily Roman Catholic masses. The number of health chaplaincy services offered daily range from one to three services, with only one service offered to patients 24 h a day.

Regarding the standards established for providing spiritual care, there is generally no legislative requirement in most healthcare facilities to provide spiritual care. In only one case is legislation prohibiting discrimination on religious grounds specified: “Yes, some legislation which forbids discrimination on religious grounds”.

This standard is possibly related to the existence of a national government policy mandating the provision of spiritual care in addition to a national nursing regulatory requirement for the provision of spiritual care: “Standards for education include reference to spiritual care”. Only if the provision of spiritual care is a component of hospital policy is there “Some reference to attending individual needs related to beliefs”.

In summary, some standards related to the expression of spirituality have been detected, but there is little consistency in care policy.

With regard to the definition of “spiritual care”, none of the healthcare centres define it concretely. In the same way, most cases in the hospital care centres did not have a requirement to document or record the personal, religious, or spiritual beliefs of patients at the time they were admitted to the hospital.

Patients’ personal, religious, or spiritual beliefs were mainly addressed at the discretion of the staff or at the patient’s own request: “Attended to at the discretion of the staff” or “Attended to at the request of the patient”.

When asked about whether nurses are given educational support to provide spiritual care, they mostly stated that they receive such education in the initial training. In other cases, nurses received educational support when they worked in palliative care: “Some education provided during training. Additional training provided for those completing postgraduate education in palliative care”.

In the totality of cases, hospital-employed healthcare chaplains are perceived as the only ones who are responsible for spiritual care in the hospital: “Hospital employed Healthcare Chaplains”.

Finally, in most healthcare facilities, there is no hospital policy for making referrals to health chaplaincy or pastoral care teams. Only some procedures exist in one case: “There are some procedures that include direct contact with the chaplain by hospital staff, patient or family”.

### 3.2. Intrapersonal Spirituality

First, the results show that nurses are generally aware of the importance of spirituality for the health and well-being of patients (Table 1). In the same vein, nurses are aware of the impact of their own values and beliefs on the provision of spiritual care. However, there is no unanimity of opinion on whether nurses are encouraged to reflect on their own personal, religious, or spiritual beliefs and to care for their personal well-being. In most cases, they stated that this does not occur.

### 3.3. Interpersonal Spirituality

In the hospital care setting, nurses mostly stated that nurses are encouraged to understand how people express their spirituality. Nurses are mostly aware of different world/religious views and how these can influence people’s responses to major life events. Finally, nursing care is respectful of people’s diverse expressions of spirituality.

### 3.4. Spiritual Care Intervention and Evaluation

In terms of how nurses identify patients’ spiritual needs, they do so primarily as part of the patient assessment through conversations they have during patient care: “Forms part of the assessment and spoken about with patients regularly during care”.

Notably, in no case did nurses use any screening tool to determine whether patients are experiencing spiritual distress. Specifically, neither they nor the chaplains make use of spiritual assessment tools such as FICA, SPIRIT, HOPE, and ETHNIC1 (S) 2Q-SAM2. However, in all cases, the healthcare team meetings did include discussions about the spirituality of the patients.

## 4. Discussion

There is an increased urge to support and educate nurses, midwives, and other healthcare workers on providing spiritual care to patients [2,48]. In this regard, spiritual care provision is the main feature of the five study sites. There is evidence of healthcare across all areas, an attribute of healthcare popularity common across many countries [49]. The COVID-19 pandemic displayed the support provided by healthcare chaplaincy, mostly when visiting was not allowed [50]. Indeed, there was an increase in demand for healthcare chaplaincy services due to the COVID-19 pandemic [51]. The high demand led to a shortage of these services in some areas [52], with special mention of better ways to provide spiritual care to patients primarily in crisis [53]. Recognized as a “collective trauma”, it is likely to have more far-reaching consequences related to the pandemic, including moral injury and psychological effects [22]. Some studies showed increased loneliness and depression among older adults locked away due to the pandemic [54]. Therefore, psychological and spiritual support is a key ongoing and future requirement for people in hospitals [22].

It was reassuring to find that nurses provide spiritual support to patients although a high proportion of patients’ lack of spiritual needs and religious affiliation was noteworthy. However, this echoes with the literature on the topic due to the lack of confidence, embarrassment, and lengthy nature of suggested assessment tools [46,55]. Experts have put forward innovative approaches, offering a simple, two-question approach (termed 2-QSAM) that prompts nurses to ask: “What’s most important to you right now?” and “How can I help?” [56]. This approach seems a key to support nurse training on spiritual care.

COVID-19 has also been challenging for nurses because it has led to the changing of healthcare roles in cases such as cancer [22]. In some cases, patients exhibited fear, with older people showing anxiety about death. Significant life challenges became magnified [30]. According to Christoph and Smith (2020), nurses experienced loneliness and isolation due to deaths during the pandemic and due to extra efforts to properly support the patients and the relatives’ grief. In this context, the correct identification of the spiritual needs of patients and relatives becomes the key to providing high-quality spiritual support [57].

A main concern is the issue of the provision of spiritual service based on a single faith, the related religious services, and the provided spaces to develop rituals or just praying, as pointed out by other work [47]. Similarly, healthcare chaplaincy based on diverse faiths is compatible with a single-faith approach [47,58]. However, this leads to the public having concerns about and being influenced by surface appearances without more in-depth information. An example of this is the presence of a chapel that has a singular connotation, which can be misleading. Multiple faiths and approaches are usual in chaplaincy services in healthcare nowadays [58]. Our Erasmus project will address nursing students’ multicultural and multifaith perspectives [45].

Another main concern is the missing education on specific competences on spiritual care for nurses [59]. In addition, there was not much training on local policy or definitions about spiritual care to offer guidance about how to offer this care, which is unrelated to other findings on the same topic [47]. Since there is an international agreement about standards on spiritual care, this lack of relationship becomes shocking [32,36,38,60]. However, this highlights the need for an imminent and structured educational action to support nurses, as pointed out by some authors [4,46,48]. This is a preference that our Erasmus Plus project will ultimately provide [61]. We are also aware that issues such as weak creativity and other skills might be compromised in our students to some extent when exclusively using virtual education [62].

Similarly, some emerging trends need consideration. In the Canadian study of Pesut et al. [31], there was a significant change in the role of the healthcare chaplain in healthcare services in terms of constraints of management and fiscal issues as well as the views from a more secularised and plural society. This has resulted in the permanent withdrawal of these services. Due to this, there needs to be more collaboration on these healthcare services from healthcare chaplains and faith leaders [63]. Spiritual care is necessary to patients and their families [64].

However, there is a lack of clarity on the practical interfacing of interdisciplinary roles. *The Lancet* in 2015 published a healthcare research series based on faith, and they could not find a reference to the duty of the healthcare chaplain; and yet, they stressed how important is to address religious and spiritual needs of patients by healthcare workers [65]. Distorting the roles can provide some conflict in healthcare, damaging significantly healthcare service by chaplains. At this point, there must be a clear definition of roles, which would benefit the implementation of future spiritual services, especially with changing demographics and non-healthcare chaplains that might provide spiritual care.

We find relevant the lack of use of digital tools in spiritual care by nurses. Indeed, the COVID-19 pandemic stressed the key part that technology can play to facilitate decentralised communication amongst patients, relatives, and healthcare staff [66], mainly for the elder, and specifically to improve psychological well-being based on “body-mind-spirit intervention” [67]. It was during the COVID-19 period that healthcare chaplains used technology for the first time [51,68]. Similarly, topics relevant to healthcare chaplains, such as death, can be very challenging with the sudden use of technology. The Erasmus Project’s proposal of using digital technology to create a base for nurses’ provision of spiritual care to patients will also come along with specific recommendations to address digital services in a sensitive way [45].

In relation to the research carried out, it goes a step further and provides specific knowledge, as it is contextualized in five EU countries, and delves into the specific needs expressed by nurses not only at a time of health crisis but in a day-to-day reality of health care, characterized by cultural diversity and an increase in the number of patients. In addition, the relationship between the spiritual, personal, and professional elements that determine spiritual care in hospitals has been shown. Finally, the results have highlighted the contribution that digital competencies can offer as a tool for continuous training for the development and knowledge of spiritual care with a professional character and adapted to the needs of each person.

## 5. Conclusions

The relevance of spirituality in supporting healthcare worldwide is well-established. Most healthcare organizations have had chaplaincy supporting patients’ religious and spiritual needs. However, healthcare chaplaincy services are not provided worldwide. There has been some disrupted or missing service because of financial cuts during the COVID-19 pandemic. Similarly, an increasing interest in considering spirituality and religion among allied health professionals can be easily found. In this regard, nurses, while consistently found to support patients’ spiritual needs, often lack specific education and training. The recent development of core competencies for nurses has created a path for the development of rigorous education in this area. The development of an online educational program by a team of international experts on digital technology will be an important contribution. We aim to provide specific education and skills to future nurses. When the nurses complete this program, they will be more sensitive to patients’ diverse spiritual needs and requirements. The nurses will also be well-equipped to support patients’ and families’ post-pandemic spiritual needs.

Four additional sections were designed and implemented based on four key competencies identified by the EPICC project as being required by active nurses. The project invited nurses to identify needs and resources from patients, to strategize engagement forums on spirituality, and to document the statistics of care [4].

These sections were focused on intra and inter-personal spirituality, assessment of spiritual care, and the related planning and evaluation of any intervention. These recommendations by the European Commission [4,32] suggest that nurses are expected to identify spiritual needs and resources, plan effective interventions, evaluate health outcomes, and document and record the process.

The researchers in this study developed a tool to collect data based on the EPICC (2021) competency framework on nursing and other related work [47]. A group of international experts, through a four-meeting series, revised, improved and wrapped up the framework; the authors also created a recommendation for a short version of the form. Notably, a competency framework formed part of the survey in this project, which provided a holistic guideline regarding spiritual care competencies, with nurses serving skilfully as a guideline for care in healthcare sites.

Ideally, the relationship that fosters interaction between a nurse and a patient and propels the nurse to be involved with the spirituality of the patient is an interpersonal relationship. However, a spirituality that awakens the awareness of one’s spirituality can also be intrapersonal.

Furthermore, the assessment is comprised of procedural input in which spiritual statistical data are obtained and the documentation conducted, while the empirical part consists of the activities carried out to address the patient’s spiritual needs. Moreover, the final component in the survey challenged the availability of up-to-date technologies platforms and networks to be used at hospital sites.

The tool was first drafted by two experts (F.T., M.C.) with the support of other experts and the project partners. The draft was inspired by previous work [47] and the abilities inspired by the project to develop competences in the provision of services, compassionate care, and education by midwives and nurses [4,32,38].

The project mentioned above eventually developed the capabilities and approaches used by the European nursing department.

The five project partners collected data in June and July 2021 in the respective countries, producing a descriptive analysis from an Excel spreadsheet.

Based on these results and considering the aspects to be addressed for spiritual care with the use of digital tools, the second phase of the project defines the digital competencies necessary for the continuous training of professionals offering spiritual care. For this purpose, the following learning matrix was established (Table 2):

## 6. Limitations

Although the findings of our study may support a large number of international initiatives on the topic, we acknowledge that our sample is quite small and has limited representation. Nonetheless, it might be used to support further research on the topic.

## 7. Implications

This research contributes to the collective construction of knowledge, motivating other researchers to advance in the field of spiritual care research, specifically in the healthcare field from a multidisciplinary approach, due to the cultural, social, professional, spiritual, and technological implications of both nursing professionals and the patients for which they care. In addition, new knowledge has been updated because of the review of the state-of-the-art, contributing to the continuity of other researchers. Finally, this work contributes to the improvement of quality in the professional practice of nurses, as the research results obtained are used in the creation of a continuous and open learning tool, through digital competencies, specially designed for the European context.

## Figures and Tables

**Table 1:** Summary of results about nurses’ approach and awareness to patients’ spirituality.

	1	2	3	4	5
Are nurses encouraged to understand how people express theis spirituality?	Yes	Yes	No	Yes	Yes
Are nurses made aware of the different world/religious views and how these may impact individuals’ responses to key life events?	Yes	Yes	No	Yes	Yes
Is nursing care respectful to individuals´ diverse expressions of spirituality?	Yes	Yes	Yes	Yes	Yes

**Table 2:** Learning matrix between digital skills and spiritual care.

Digital Skills for Spiritual Attention	Spiritual and Care in Hospital
Digital support to promote the spirituality of nurses	Frenemies: spirituality and religios
	Spirituality and care, and spirituality in care around the word
	Spirituality in healthcare settings
	Tye spirituality of care professionals and nurses
	Research tools for the study of patientients´spiritual needs
Spirituality and care and spirituality in care around the world—mapping, networking, and metiation	**Managing religious diversity**
	Christianity: Cotholicism, Protestantism, and Orthodoxy
	Judaims and Islam
	Buddhism, Hiduism, and Sikhism
	Jehovah´s Witnesses, agnosticism, and atheism
Organising and managing information to promote spirituality and care of nurses	**Narrative medicine**
	Narrative-based medicine
	Narrative medicine and spirituality: the professional point of view
	Narrative medicine and spirituality: patiens, family members, and dialogues
	Narrative medicine: measurement and evaluation
	Narrative medicina and spirutality beyond sciencitific papers

## Data Availability

Available under request to the corresponding author.
